# Prognostic factors in early-stage ovarian cancer

**DOI:** 10.3332/ecancer.2013.325

**Published:** 2013-06-13

**Authors:** Germana Tognon, Mario Carnazza, Monica Ragnoli, Stefano Calza, Federico Ferrari, Angela Gambino, Valentina Zizioli, Sara Notaro, Benedetta Sostegni, Enrico Sartori

**Affiliations:** 1 Department of Obstetrics and Gynecology, Spedali Civili di Brescia, Italy; 2 Department of Molecular and Translational Medicine, University of Brescia, Italy

**Keywords:** early stage ovarian cancer, prognostic factors, peritoneal cytology, dense adhesions, adjuvant chemotherapy, taxanes, conservative treatment

## Abstract

The purpose of this study was to identify the main prognostic factors in patients with early-stage epithelial ovarian cancer. Data were extracted from 222 patients with initial stage (I–IIA) invasive epithelial ovarian cancer treated with primary surgery followed or not followed by adjuvant therapy, from 1 January 1980 to 31 December 2008, at the Division of Obstetrics and Gynecology, Spedali Civili, Brescia, Italy; the median follow-up was 79 months (SD ± 35,945, range 20–250 months).

The negative prognostic factors that were statistically significant (p<0.050) in univariate analysis were grade 2, 3, and X (clear cell in our study); stage IB, IC, IIA; positive peritoneal cytology, age equal to/greater than 54; dense adhesions; capsule rupture (pre-operative or intra-operative) and endometrioid histotype (only for disease-free survival (DFS)). Positive cytology was strongly associated with peritoneal relapses, while adhesions were associated with pelvic relapses. A positive prognosis was associated with the mucinous histotype.

Conservative treatment had been carried out in 52% of patients under 40 years of age, and we detected only two relapses and three completions of surgery after a few weeks among 31 women in total. Our study indicated a possible execution in patients with patients with cancer stage IA G1–G2 (p=0.030) or IC G1 (p=0.050), provided well staged. Adjuvant chemotherapy improved the survival of cancers that were not IA G1. The positive prognostic role of taxanes must be emphasised, when used in combination with platino.

## Introduction

Ovarian cancer is usually diagnosed at an advanced stage. Nevertheless, about 30% of patients present with early-stage disease (FIGO stage I–IIA). Surgery is the mainstay in the treatment of epithelial ovarian cancer, and an extensive surgical staging is fundamental in the selection of most appropriate post-surgical therapy, requiring the removal of all macroscopic tumour, along with total abdominal hysterectomy, bilateral salpingo-oophorectomy, and infracolic omentectomy. Any ascitic fluid should be removed or, alternatively, peritoneal washing should be collected. If disease appears confined to the ovary, biopsies of the diaphragmatic peritoneum, paracolic gutters, pelvic peritoneum, and sampling or dissection of paracolic and pelvic nodes are required to make optimal staging [12].

In spite of the generally favourable outcome of early-stage ovarian cancer, there is considerable heterogeneity with respect to the risk of relapse, ranging from 15% to over 40% in various reports [2, 6, 7, 30].

It is, therefore, important to identify the risk factors of relapse in order to adapt risk-tailored strategies to improve outcomes. Several factors, such as age, stage, grade, histological subtype, ascites, intra-operatorial adhesions, and cyst rupture, have been identified as potential risk factors. There are numerous biases concerning early-stage ovarian cancer studies, such as sample size [31, 32], inclusion of borderline tumours [7], inadequacy of surgical staging [6], presence of microscopic metastasis not initially recognised [18, 19], and collection of data from many countries [2].

The aim of our study is to define the significant prognostic factors in early-stage ovarian cancer by a detailed univariate and multivariate statistical analysis of a well-documented group of patients.

## Method

The study includes 222 optimally staged patients with early-stage (I–IIA) epithelial ovarian cancer treated with surgery followed or not by chemotherapy, from 1 January 1980 to 31 December 2008, at the Division of Obstetrics and Gynecology, Spedali Civili, Brescia, Italy.

Overall survival (OS) and disease-free survival (DFS) times were defined as the period between primary surgery and death or relapse, respectively. Patients dying of intercurrent disease were censored at the time of death when the actuarial DFS was calculated.

The cumulative survival rate was calculated by the Kaplan–Meier method. The independent effects of prognostic factors and other covariates on survival function were determined by Cox’s proportional-hazard regression models. Significance was defined as a p<0.050.

## Results

Patients with a median follow-up of ten years showed ten-year OS and RFS of 75% and 74%, respectively.

Fertility-preserving surgery was acceptable, provided that full surgical staging had been carried out. This kind of surgery was performed in 31 patients, 26 of these under the age of 40, and we detected only two pelvic relapses, more precisely one tumour IC G2 mucinous and one tumour IC clear cell. Neither of these two relapses were found in the contralateral ovary. The other three patients with tumour IA G2, initially subjected to conservative surgery, were subsequently subjected to a second surgery a few weeks after the completion of adjuvant chemotherapy in order to eliminate the residual tumour. The remaining 26 patients (seven treated with adjuvant chemotherapy and 19 without) did not develop relapse during regular follow-up, and at predictive analysis, conservative therapy can be considered in patients with tumour IA G1–2 (p=0.030) and even IC G1 (p=0.050), provided well staged. Adjuvant chemotherapy did not improve the prognosis of patients treated conservatively. We observed 18 pregnancies completed successfully.

Forty-six patients received systematic pelvic and lomboaortic lymphadenectomy to improve surgical staging, and we found it was associated with the best OS and DFS, although this was not statistically significant; in a predictive analysis, only G3 tumours benefited significantly (p=0.030) from this procedure.

The second-look procedure does not show a positive prognostic role, which may be due to the bias in the selection of patients undergoing the procedure: in fact, 71% of them belonged to the high risk group (IC–IIA).

Even adjuvant chemotherapy, analysed in patients staged other than IA G1, was an important factor that improved survival, although not statistically significant, due to the lack of efficacy (p>0.050) in the group of cancers well differentiated (G1 IB–IC–IIA) or IA moderately differentiated (G2).

Ninety-six percent of patients had platinum-based chemotherapy, and 88% of them used it for relapse treatment; this confirms its prominent role in chemotherapy for ovarian cancer. For this reason, it has not been possible to analyse its prognostic role from a statistical point of view. The percent of patients who used it as a single agent is 30.7, while the percent of patients who used it in combination with taxanes or cyclophosphamide is 69.3.

The introduction of taxanes in patients treated with adjuvant chemotherapy appears essential. In particular, this drug, administrated to 45 patients with platinum, had a positive effect on the OS and DFS in univariate analysis; this was confirmed in multivariate analysis only for DFS.

Only 12% of patients receiving chemotherapy developed significant toxicity.

Adjuvant therapy changed over the years between 1980 and 2008, in particular with the introduction of taxanes in combination with platinum drugs in 1997.

In the 1980s, we observed a 27.5% recurrence rate. During those years, standard adjuvant chemotherapy was represented by cisplatin, alone or in combination with cyclophosphamide. The use of a single adjuvant agent showed a negative prognostic role (p<0.050) in comparison with combined chemotherapy. Only three patients during this decade achieved adjuvant radiotherapy without simultaneous or successive chemotherapy, so no conclusion can be drawn about the comparison of these two adjuvant treatments.

In the 1990s, the recurrence rate was 20%. During this decade, single-agent adjuvant therapy (carboplatin or cisplatin) assumed a negative prognostic role (p<0.050) against combined-agent therapy (cisplatin with cyclophosphamide or, from 1997, carboplatin with paclitaxel).

During the 2000s, we have observed a 12% rate of recurrence. In this decade, a combination of carboplatin with paclitaxel confirmed its role as standard adjuvant chemotherapy for early-stage ovarian cancer. Among the patients with ovarian cancer that was not IA G1, 17 did not receive adjuvant chemotherapy in the 1980s as compared to only six in the 2000s. This reflects a better understanding of the importance of adjuvant chemotherapy in the treatment of early-stage ovarian cancer and its systematic use.

**Figure 1:**
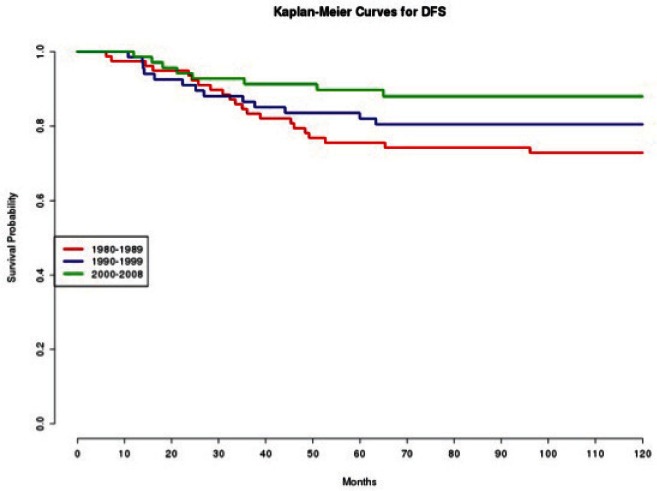
The DFS of patients according to the decade of treatment (1980–1989; 1990–1999; 2000–2008).

**Figure 2:**
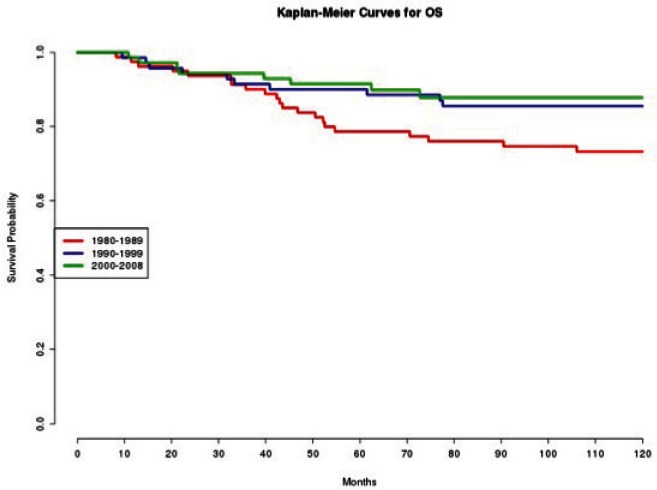
The OS of patients according to the decade of treatment (1980–1989; 1990–1999; 2000–2008).

In effect, we observe how patients treated in the 1980s had a statistically significant (p=0.020 and p=0.010, respectively) worse DFS (Figure 1) and OS (Figure 2), compared with those treated in the 2000s.

Table 1 resumed characteristics of cancers in terms of DFS ten-year survival.

We can notice how ten-year global survival for patients involved in the study was 74% and 75% for DFS and OS, respectively. As in earlier studies [7, 25, 26], DFS was the first endpoint and OS the second endpoint to establish the prognostic value of the different variables.

In this study, the combination of stage and grade stratified patients into three groups with significantly different prognoses: a low-risk group represented by tumour stage IA–IB G1, a moderate-risk group represented by tumour stage IA–IB G2–3-X, and finally a high-risk group with the worst prognosis, represented by IC–IIA tumours (Table 3).

**Figure 3:**
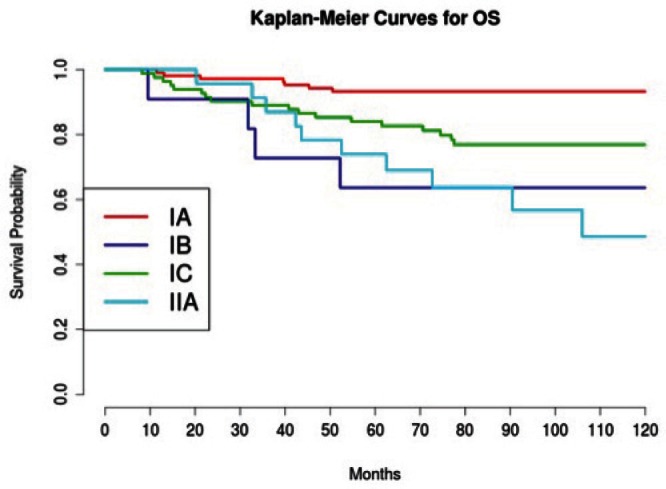
The OS of patients according to stage.

Considering 1988 FIGO stage classification, we reported a poor prognosis for patients with IB and IIA cancers (Figure 3); the results were, however, affected by the low number of cases detected, 11 and 23, respectively. On the other hand, stage IC was not a significant negative prognostic factor in multivariate analysis (Table 4 and Figure 3). This implied low prognostic importance for surface excrescences (Table 2), present in 65% of stage IC tumours, and at the same time, was strongly correlated to the detection of a statistically significant negative prognostic value for capsule rupture only at univariate analysis (Table 3). Only four of 48 cyst ruptures occurred during surgery, so no conclusion can be drawn from that. There is no evidence that capsule rupture during surgery may promote metastases. However, the negative significance of capsule rupture reported at univariate analysis should induce surgeons to avoid rupture during surgery. The four ruptures during surgery were observed in patients who underwent laparotomy; therefore, no firm conclusions can be made for the endoscopic removal of malignant tumours confined to the ovaries. In view of the reports [27–29] on rapid peritoneal spread after laparoscopic removal of ovarian cancer, an unexpected malignant lesion found at laparoscopy and documented by frozen-section histopathological analysis should require an immediate staging laparotomy.

**Figure 4:**
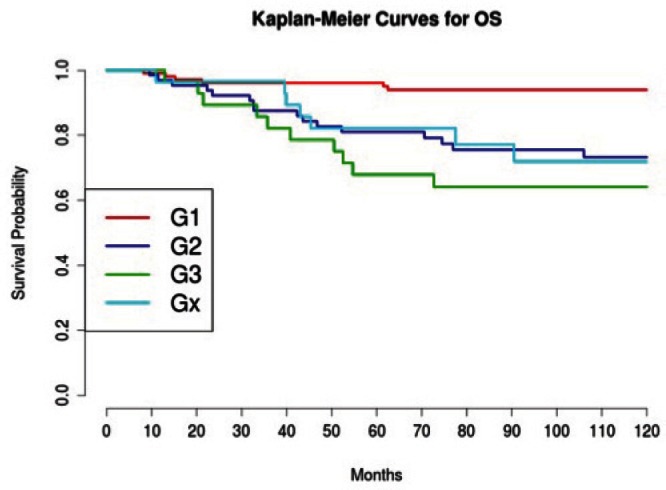
The OS of patients according to grading.

**Figure 5:**
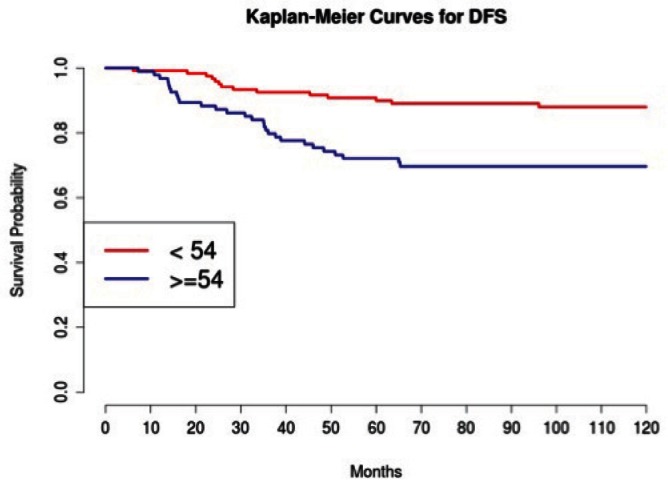
The DFS of patients in consideration of age less than or greater than 54 (average age 53.7).

**Figure 6:**
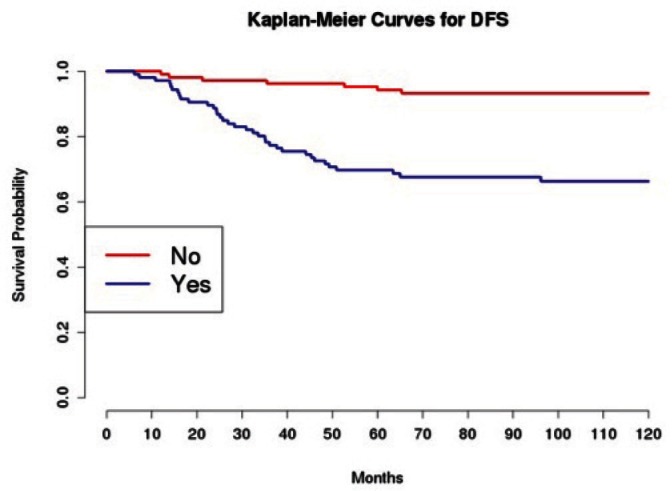
The DFS of patients based on the presence of intra-operatory adhesions.

**Figure 7:**
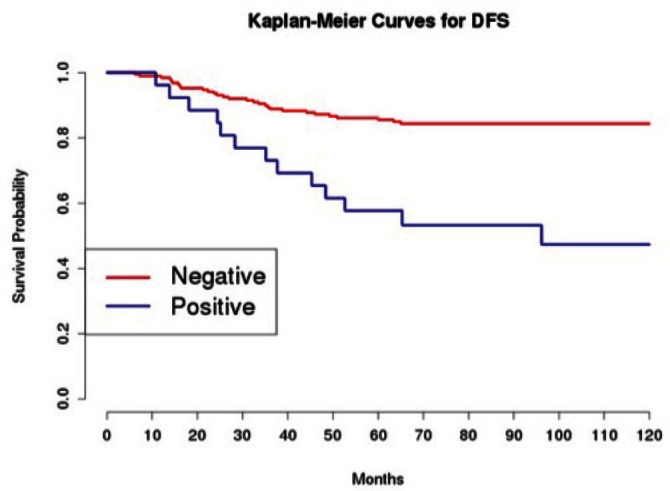
The DFS of patients based on the presence of positive peritoneal cytology.

**Figure 8:**
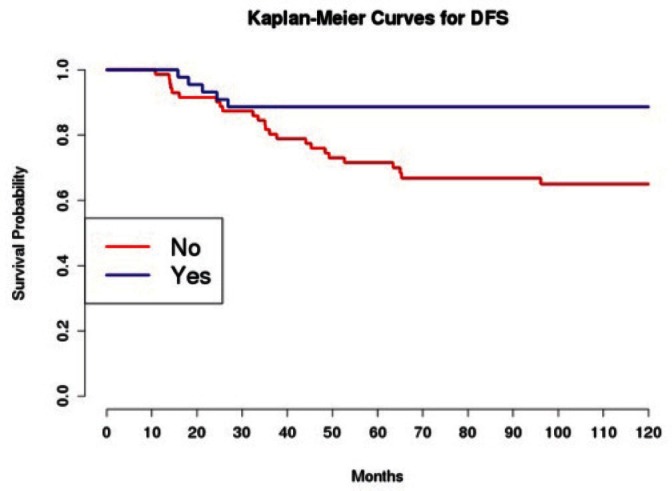
The DFS of patients treated with taxanes among those receiving adjuvant chemotherapy.

Grading was a stronger prognostic factor than stage at predictive analysis (p=0.030), but its value on multivariate analysis was confirmed only for OS (Figure 4). Age (Figure 5), dense adhesions (Figure 6), positive peritoneal cytology (Figure 7), and use of taxanes (Figure 8) were the most powerful predictors of relapse. The prognostic role for stage, degree of differentiation, positive peritoneal cytology, and dense adhesions has been confirmed, allowing for a decade-specific (1980s,1990s,2000s) analysis.

In our study, 97 patients with an age equal to or greater than 54 (average value 53.7) were associated with statistically significant worse prognoses in univariate and multivariate analysis.

Our results were consistent in finding positive peritoneal cytology and intra-operatory dense adhesions as negative prognostic factors at univariate and multivariate analysis (Tables 3 and 4) (the value for multivariate adhesions was confirmed only for DFS). In particular, positive cytology, reported in 28 patients, was strongly associated with peritoneal relapses (eight recurrences with positive cytology values of 13), while dense adhesions were associated with pelvic relapses (91% of relapses).

No difference can be made between the importance of ascites, defined as peritoneal effusion that in the opinion of the surgeon was pathological or clearly exceeded normal amounts (50 ml), and positive cytology, due to the close coincidence of these factors, with only three patients with positive cytology washing without ascites and two patients with ascites without tumoural cells on peritoneal cytology.

The prognostic importance of pre-operative CA 125, CA 19-9, CA 15-3 (lower or equal to/greater than 60 UI/ml, mean value noticed) was not statistically significant.

Regarding the role of histotype, mucinous tumours had the best prognosis, but this positive value was not confirmed at multivariate analysis. Similarly, endometrioid tumours were associated with worse prognosis (statistically significant only for DFS) but not confirmed at multivariate analysis. It must be underlined, however, that 54% of mucinous tumours belonged to the low-risk group (IA–IB G1), while 54.2% of endometrioid tumours belonged to the high-risk group (IC–IIA).

Predictive analysis of relapses showed a statistically significant role (p<0.050) for stages IB, IIA; G2, G3, GX; age equal to/greater than 54; dense adhesions; and positive cytology; this is in agreement with the results of multivariate analysis (Table 3).

## Discussion

Our study showed the great importance of classic prognostic factors like stage (only IB and IIA) and degrading, in concordance with conclusions reported by Dembo *et al *[1] and Vergote [2]. It was also shown that parameters such as positive cytology and intra-operatory adhesions, defined in our study, agree with Dembo *et al *[1] as those in which sharp dissection was needed, a raw or oozing area was left, cyst rupture resulted from dissection of the adherence or direct tumour invasion of adjacent structures was observed.

Positive cytology and adhesions were, in many studies, strongly connected with the tumour’s metastatic potential, and with a particular molecular arrangement, in particular loss of e-cadherin [39], positive of mib-1 and p-53 [33], aneuploidy of DNA [34, 35, 36] and mitotic activity index (MAI) [37]. An attractive hypothesis is highlighting possible alternative characteristics of these tumoural cells such as a reduced mutual adhesiveness [39], a reduced contact inhibition movement [40, 42], and a mobility increase compared with other tumour cells [38]. The reduced mutual adhesiveness has precise ultrastructural bases, reduction of membrane’s invaginations and evaginations that connect the cell to similar cells [41], and chemical–physical characteristics related to the constitution of the membrane and the pattern of distribution of various surface constituents that results in modification of the charge electronegative distribution and therefore of surface code [40, 41, 42]. Mutual contact inhibition is due to surface abnormalities, while the increased mobility could be caused by abnormalities in the cytoskeleton [41]. These considerations highlight the importance of positive cytology and adhesions for DSF, in particular.

Age appears to be another factor of extraordinary importance. Hormonal status certainly plays a key role, but the association of a young age for a woman with an immune response capable of tackling and countering cells with mutations and extraordinary reproductive capacity remains a very important factor [44].

It is important to underline the modifications of the immune system with age [45]: there is a decrease in the number of T cell effectors, a decrease in the number of functional antibodies, and especially, in the ability to effectively respond to foreign antigens. Once a mutated cell is recognised, the numerous effectors of immune and cell-mediated response in the young patients are able to destroy or at least greatly limit the metastatic potential of malignant cells, and in this case, a major role seems to be carried out by lymphocyte T cytotoxic. That age is a strong prognostic value agrees with conclusions of many studies on ovarian cancer, particularly those reported by Smedley [3], Thigpen [4], and Duska [5].

The positive role of mucinous tumours seems to be correlated with a low potential metastatic activity, with a major response to adjuvant chemotherapy and with the assumption of great size, so that cancer can produce a higher frequency of symptoms and signs at presentation, resulting in earlier detection. Another argument for mucinous tumours to remain localised until they become pelvic cysts is the hypothesis that mucinous tumours remain attached to the mesothelial layer, whereas serous and endometrioid tumours invade rapidly into this cell layer [46]. A negative prognostic role for endometrioid tumours has not been previously noticed and appears strongly connected with the high percentage (54.2%) of high-risk (IC–IIA) tumours with this histotype. These considerations agree with those reported by Vergote [2], Chan [6], and Ahmed [7].

Tumour markers did not have important prognostic role in this study; their value appears to be most significant in the screening of high-risk women and in the follow-up of patients subjected to primary therapy, particularly after adjuvant chemotherapy. In fact, there was great sensitivity (70%) in our study in predicting relapse. In 45% of cases, it was the first investigation to put a suspicion on recurrence. Our conclusions are concordant with those of Sevelda [8], Makar AP [9], Osman [10], and Buller [11].

Our study took into consideration patients’ optimal stage. In early ovarian cancer, staging was of extraordinary importance to avoid maintaining microscopic residual tumours and to adequately highlight peritoneal and lymph nodal metastatic foci. Zanetta [12] reported an important study of 351 patients with stage I ovarian tumours, in which at multivariate analysis only grade (1–2 versus 3) and staging were statistically significant prognostic factors.

Our results about the feasibility of conservative surgery agree with the conclusions of Zanetta [13] *et al *in their retrospective analysis of 99 women, aged under 40, treated between 1982 and 1993. In their series, 56 patients received fertility-sparing surgery, while 43 were treated with the removal of the internal genital apparatus. After a median follow-up of seven years, the authors report a 9% recurrence rate in the group treated conservatively versus 12% in those treated with ablative surgery. The low (3.5%) rate of contralateral recurrence justifies the conservative approach and either because the rate of unrecognised metastases in the contralateral ovary was lower than generally expected or because adjuvant chemotherapy might eradicate microscopic neoplastic foci, the preservation of the internal genitus apparatus did not affect the possibility of curing the disease. Their conclusion was therefore that conservative treatment may be safe in women with early-stage ovarian cancer, even for some stage IC disease, provided that optimal stage and informed consent are achieved.

When using a conservative approach, special attention should be paid to the contralateral gonad. According to our results, with no relapse in the remaining ovary clinically free, a careful inspection of the ovarian surface seems to be adequate, thus avoiding inappropriate wedge biopsies or even multiple surface biopsies that might impair the future reproductive function through a compromised vascularisation of the ovarian hilus.

Concerning systematic lymphadenectomy, our results in the moderate–high risk group (non-IA–IB G1) agree with those by Suzuki [24], Lang [16], and Onda [17], who reported an important contribution in improving staging avoiding microscopic lymphonodal metastasis. These authors reported a role for systematic lymphoadenectomy, especially for serous and G2, G3 cancer. We found a significant role (p=0.020) only for low differentiated tumours. On the other hand, a multicentre, prospective, randomised Italian study from Benedetti Panici [15] showed that an extensive procedure in the retroperitoneal area demands a formidable surgical effort, carrying distinct risks and morbidity and a longer operating time and hospital stay. Moreover, its therapeutic value is controversial, and in the Italian study, it seems to have no value. Italian data are in accordance with the previous retrospective experience of Petru *et al *[14], reporting no absolute difference in five-year survival of patients undergoing a radical lymphoadenectomy (82%) versus no lymphoadenectomy (87%).

The benefits noticed from adjuvant chemotherapy, except for patients IA G1, for which it is not recommended, agreed with the conclusions of the two great trials ICON-1 [18] and EORTC ACTION [19]; according to reports, chemotherapy is the best form of therapy after primary surgery. Before these two great trials, even Bolis [22], in his study, reported an important advantage in terms of survival for patients treated with chemotherapy, while conclusions in a Scandinavian study [23] published in 2000 did not report any kind of advantage between adjuvant chemotherapy and observation after primary surgery.

Our results about positive role of taxanes in combination with platinum agree with the results reported for paclitaxel and cisplatin in the GOG-111 [20] and European Canadian Intergroup (OV10) [21] trials, both of which compared paclitaxel plus cisplatin with cisplatin plus cyclophosphamide.

A time-specific analysis detects how, in the 1980s, standard adjuvant chemotherapy for ovarian cancer consisted of cisplatin, used alone or in combination with cyclophosphamide. Among these patients, the combination assumed a positive prognostic role, considering the synergic mechanism of action of these drugs. These results agree with those reported by Decker Dg *et al *[47], concerning the best treatment programme for advanced stage ovarian cancer. The same statistically significant positive prognostic value (p<0.050) for the combination versus single agent adjuvant chemotherapy was also found in the group of patients treated in the 1990s. To underline the use of the combination paclitaxel–carboplatin, it is the standard adjuvant chemotherapy of ovarian cancer since 1997. The better prognoses for patients treated in the 2000s versus those treated in the 1980s depends on the introduction, in 1997, of taxanes and on the systematic use of adjuvant chemotherapy for patients with early-stage ovarian cancer, excluding IA G1.

Concerning the second look in the moderate–high risk group (non-IA–IB G1), its value seems to be low in the correct management of early-stage ovarian cancer, unlike in advanced-stage cancer [48], particularly considering the innovations of new diagnostic techniques such as TAC or PET used for the study of neoplastic patients with suspicious relapse.

## Figures and Tables

**Table 1:** Ten-year survival in relation to various characteristics of patients.

Characteristics	Number	Percentage	Ten-year DFS (%)
**Grading**			
Good	101	46%	92
Moderate	64	28.8%	65
Poor	28	12.6%	60
Not graded	28	12.6%	76
**FIGO Stage 1988**			
IA	102	46%	91
IB	11	5%	67
IC	82	36.9%	72
IIA	23	10.4%	46
**Risk Group**			
Low risk IA–IB G1	71	32%	91
Moderate risk IA–IB G2–G3–GX	46	20.7%	80
High risk IC–IIA	105	47.3%	62
**Histological type**			
Serous	71	32%	78
Mucinous	63	28.4%	84
Endometrioid	48	21.2%	65
Clear cell	28	12.6%	73
Mixed epithelial	8	3.6%	76
Other	4	1.8%	79
**Dense adhesions**			
Yes	108	48.9%	61
No	114	51.1%	90
**Peritoneal cytology**			
Yes	28	12.6%	43
No	194	87.4%	81
**Capsule rupture**			
Yes	48	21.4%	66
No	174	87.4%	78
**Age at diagnosis**			
<54	125	56.3%	84
>54	97	43.7%	66
**Pre-operatory CA125**			
<60 UI/ml	77	54.6%	85
>60 UI/ml	64	45.4%	76
**Patients non-IA G1**			
Treated *with* chemotherapy	113	72.9%	69
*With taxanes*	*45*	*37.8%*	*84*
*Without taxanes*	*74*	*62.2%*	*60*
Treated *without* chemotherapy	42	27.1%	65
**Lymphoadenectomy in moderate–high risk group**			
Yes	41	27.1%	76
No	110	72.9%	62
**Second look in moderate–high risk group**			
Yes	47	31.2%	58
No	104	68.8%	74

**Table 2:** Characteristics of stage IC patients.

Characteristics	Number	Percentage
**Positive cytology**	28/81	34.1%
**Ascites**	27/81	32.9%
**Capsule rupture**	48/81	58.5%
**Surface excrescenses**	57/81	69.5%

**Table 3:** Actuarial DFS and OS related to the most important prognostic factors in early-stage ovarian cancer byunivariate analysis.

Characteristics	DFS			OS		
	HR	p value	95% CI	HR	p value	95% CI
**Degree of differentiation**						
G2 versus G1	6.4	0.001	2.5–15.995	4.3	0.002	1.8–10.4
G3 versus G1	9.02	0.001	3.3–24.4	8.36	0.001	3.2–21.4
GX versus G1	5.016	0.004	1.7–14.9	4.93	0.003	1.8–13.6
**FIGO stage 1988**						
IB versus IA	5.3	0.002	1.4–20.1	8.9	0.001	2.9–27.57
IC versus IA	3.9	0.001	1.7–8.9	3.9	0.002	1.7–8.7
IIA versus IA	9.1	0.003	3.7–22.4	7.4	0.000	2.9–18.9
**Group risk**						
IA–IB G2–3-X versus IA–IB G1	4.7	0.002	2–10.1	5.7	0.002	2.8–11.7
IC–IIA versus IA–IB G1	9.1	0.002	4.5–18.7	10.1	0.002	4.2–19.5
**Age**						
>54 versus <54	2.9	0.002	1.5–5.4	2.3	0.008	1.2–4.2
**Peritoneal cytology**						
Yes versus No	3.6	0.010	1.9–6.9	3.1	0.001	1.5–5.9
**Capsule rupture**						
Yes versus No	2.0	0.030	1.0–3.7	1.9	0.040	1.0–3.6
**Dense adhesions**						
Yes versus No	5.4	0.001	2.5–11.7	3.5	0.010	1.7–6.9
**Histological type**						
Mucinous versus other	0.4	0.040	0.2–0.9	0.4	0.040	0.2–0.9
Serous versus other	0.86	0.660	0.4–1.6	0.95	0.800	0.5–1.7
Endometrioid versus other	1.9	0.048	1–3.5	1.7	0.110	0.8–3.2
Clear cell versus other	1.5	0.200	0.7–3.2	1.6	0.200	0.8–3.3
**Pre-operatory CA125**						
>60 versus <60 UI/ml	1.7	0.200	0.7–4	1.5	0.300	0.5–3.6
**Adjuvant chemotherapy in moderate–high risk group (non-IA G1)**						
Yes versus No	0.79	0.500	0.4–1.5	0.6	0.170	0.3–1.2
**Taxanes in patients treated with chemotherapy**						
Yes versus No	0.3	0.016	0.1–0.8	0.4	0.063	0.1–1.05
**Lymphoadenectomy in moderate–high risk group (non-IA–IB G1)**						
Yes versus No	0.68	0.300	0.3–1.4	0.66	0.300	0.3–1.4
**Second look in moderate–high risk group (non-IA–IB G1)**						
Yes versus No	1.9	0.060	1–3.6	2.1	0.050	1.1–4

**Table 4:** Significant variables for actuarial DFS and OS in final multivariate model.

Characteristics	DFS		
	HR	p value	95% CI
**Age**			
>54 versus <54	2.6	0.027	1.2–5.8
**Peritoneal cytology**			
Positive versus negative	1.9	0.040	0.8–4
**Dense adhesions**			
Yes versus No	4.7	0.004	1.6–13.6
**Taxanes**			
Yes versus No	0.26	0.009	0.1–07

	OS		
	HR	p value	95% CI
**Grading**			
G2 versus G1	4.5	0.002	1.7–11.6
G3 versus G1	5.1	0.003	1.7–15.1
Gx versus G1	4.8	0.007	1.5–15.2
**Age**			
>54 versus <54	2.8	0.003	1.4–5.4
**FIGO s1988**			
IB versus IA	6.3	0.006	1.7–23.8
IC versus IA	1.2	0.078	0.7–2.3
IIA versus IA	3.7	0.002	1.2–11.2
**Peritoneal cytology**			
Positive versus negative	3.3	0.008	1.3–8.1
